# Two Monotonicity Results for Beta Distribution Functions

**DOI:** 10.3390/e26110938

**Published:** 2024-10-31

**Authors:** Kurt Hornik

**Affiliations:** Institute for Statistics and Mathematics, WU Wirtschaftsuniversität Wien, Welthandelsplatz 1, A-1020 Wien, Austria; kurt.hornik@wu.ac.at

**Keywords:** Beta distribution, Gamma distribution, monotonicity

## Abstract

Write pbeta(·,α,β) for the distribution function of the Beta distribution with parameters α and β. We show that α↦pbeta(α/(α+β),α,β) is decreasing and α↦pbeta(α/(α+β),α+1,β) is increasing over the positive reals, with the common limit for α→∞ expressible in terms of the Gamma distribution functions, and discuss implications for the distribution functions of the Gamma, Poisson and Binomial distributions.

## 1. Introduction

Write Gamma(α) and Beta(α,β) for, respectively, the Gamma distribution with shape parameter α and rate/scale parameter 1 and the Beta distribution with parameters α and β, and write pgamma(·,α) and pbeta(·,α,β) for the corresponding cumulative distribution functions.

If $X_{\alpha }∼\mathrm{Gamma}\left(\alpha \right)$, $E(X_{\alpha }/\alpha )=1$. Using the addition theorem for the Gamma distribution, as α→∞, we have $X_{\alpha }/\alpha \to 1$ by the law of large numbers and $P\left(X_{\alpha }/\alpha \leq 1\right)=\mathrm{pgamma}\left(\alpha ,\alpha \right)\to 1/2$ by the central limit theorem. On the other hand, using integration by parts, for ϵ>0 we have

$$P\left(X_{\alpha }/\alpha >\epsilon \right)=\frac{1}{\Gamma (\alpha )}\int_{\epsilon \alpha }^{\infty }t^{\alpha -1}e^{-t}\;dt=\frac{1}{\Gamma (\alpha +1)}\int_{\epsilon \alpha }^{\infty }t^{\alpha }e^{-t}\;dt-\frac{{\left(\epsilon \alpha \right)}^{\alpha }e^{-\epsilon \alpha }}{\Gamma (\alpha +1)}$$

so that as α→0+

$$P\left(X_{\alpha }/\alpha >\epsilon \right)\to \frac{1}{\Gamma (1)}\int_{0}^{\infty }e^{-x}\;dx-1=0$$

and hence, in particular, $P\left(X_{\alpha }/\alpha \leq 1\right)=\mathrm{pgamma}\left(\alpha ,\alpha \right)\to 1$.

Write

$$\pi_{\alpha }=P\left(X_{\alpha }/\alpha \leq 1\right),\;X_{\alpha }∼\mathrm{Gamma}\left(\alpha \right).$$

Ref. [1] shows that $\alpha \mapsto \pi_{\alpha }=\mathrm{pgamma}\left(\alpha ,\alpha \right)$ decreases monotonically from 1 to 1/2 as α varies from 0 to *∞*. In this paper, we show that, quite remarkably, monotonicity continues to hold if 1 is replaced by the normalized Gamma distributed random variable $X_{\beta }/\beta$ independent of $X_{\alpha }/\alpha$. We also establish a second, related monotonicity result. These results are formulated and discussed in Section 2. Section 3 gives the proofs.

## 2. Results

**Theorem** **1.** *For α,β>0, let $X_{\alpha }∼\mathrm{Gamma}\left(\alpha \right)$ be independent from $X_{\beta }∼\mathrm{Gamma}\left(\beta \right)$ and let $Y_{\alpha ,\beta }∼\mathrm{Beta}\left(\alpha ,\beta \right)$ so that $E\left(Y_{\alpha ,\beta }\right)=\alpha /\left(\alpha +\beta \right)$. Write*

$$p_{\alpha ,\beta }=P\left(X_{\alpha }/\alpha \leq X_{\beta }/\beta \right).$$

*Then*

$$p_{\alpha ,\beta }=P\left(Y_{\alpha ,\beta }\leq E\left(Y_{\alpha ,\beta }\right)\right)=\mathrm{pbeta}\left(\frac{\alpha }{\alpha +\beta },\alpha ,\beta \right),$$

*the function $\alpha \mapsto p_{\alpha ,\beta }$ is decreasing for α>0, with limits 1 for α→0+ and 1−pgamma(β,β)<1/2 for α→∞, and the function $\beta \mapsto p_{\alpha ,\beta }$ is increasing for β>0, with limits 0 for β→0+ and pgamma(α,α)>1/2 as β→∞.*

The above can also be formulated in terms of the Beta prime and *F* distributions, noting that trivially

$$p_{\alpha ,\beta }=P\left(\frac{X_{\alpha }}{X_{\beta }}\leq \frac{\alpha }{\beta }\right)=P\left(\frac{X_{\alpha }/\alpha }{X_{\beta }/\beta }\leq 1\right)$$

where $X_{\alpha }/X_{\beta }$ has a Beta prime distribution with parameters α and β, and $\left(X_{\alpha }/\alpha \right)/\left(X_{\beta }/\beta \right)$ has an *F* distribution with parameters α/2 and β/2. Note also that for β→∞, $X_{\beta }/\beta \to 1$ in probability and, thus, $p_{\alpha ,\beta }\to P\left(X_{\alpha }\leq \alpha \right)=\mathrm{pgamma}\left(\alpha ,\alpha \right)$, so Theorem 1 implies the result of [1].

Refs. [2,3] show that pbeta(α/(α+β),α+1,β)<1/2 for, respectively, all positive integers or all positive reals α,β. The following substantially improves these results:

**Theorem** **2.** *Let β>0. The function*

$$\alpha \mapsto {\tilde{p}}_{\alpha ,\beta }=\mathrm{pbeta}\left(\frac{\alpha }{\alpha +\beta },\alpha +1,\beta \right)$$

*is increasing for α>0, with limits 0 for α→0+ and 1−pgamma(β,β) for α→∞.*

If we write pbinom(·,n,p) for the cumulative distribution function of the Binomial distribution with parameters *n* and *p*, then for integer 0≤k≤n we have

pbinom(k,n,p)=pbeta(1−p,n−k,k+1).

Using Theorem 1 with α=n−k, β=k+1 and 1−p=α/(α+β)=(n−k)/(n+1), we obtain for integer 0≤k<n

$$\mathrm{pbinom}\left(k,n,\frac{k+1}{n+1}\right)\geq 1-\mathrm{pgamma}\left(k,k\right).$$

Similarly, using Theorem 2 with α+1=n−k, β=k+1 and 1−p=α/(α+β)=(n−k−1)/n, we obtain for integer 0≤k<n−1

$$\mathrm{pbinom}\left(k,n,\frac{k+1}{n}\right)\leq 1-\mathrm{pgamma}\left(k,k\right).$$

If $X_{\alpha +1}∼\mathrm{Gamma}\left(\alpha +1\right)$ is independent from $X_{\beta }∼\mathrm{Gamma}\left(\beta \right)$, $X_{\alpha +1}/\left(X_{\alpha +1}+X_{\beta }\right)∼\mathrm{Beta}\left(\alpha +1,\beta \right)$, so that

$$\mathrm{pbeta}\left(\frac{\alpha }{\alpha +\beta },\alpha +1,\beta \right)=P\left(\frac{X_{\alpha +1}}{X_{\alpha +1}+X_{\beta }}\leq \frac{\alpha }{\alpha +\beta }\right)=P\left(X_{\alpha +1}\leq \alpha \frac{X_{\beta }}{\beta }\right).$$

For β→∞, $X_{\beta }/\beta \to 1$ in probability, so Theorem 2 yields the following:

**Corollary** **1.** *The function α↦pgamma(α,α+1) is increasing for α>0, with limits of 0 for α→0+ and 1/2 for α→∞.*

In combination with [1] (or using Theorem 1 with β→∞), we, thus, find that the median $m_{\alpha }$ of Gamma(α) satisfies $\alpha -1<m_{\alpha }<\alpha$, where the lower bound is worse than the sharp lower bound $m_{\alpha }>\alpha -1/3$ of [4].

If we write ppois(·,λ) for the cumulative distribution function of the Poisson distribution with parameter λ, then for the integer n≥0 we have

ppois(n,λ)=1−pgamma(λ,n+1).

From Corollary 1, we, thus, find that n↦ppois(n,n)=1−pgamma(n,n+1) is decreasing from 1 to 1/2 as *n* varies over the non-negative integers, nicely extending the bounds of [5] in the integer case. We note that λ↦ppois(λ,λ) is not monotone: it jumps at the positive integers, and for n≤λ<n+1

ppois(λ,λ)=ppois(n,λ)=1−pgamma(λ,n+1)

which decreases from ppois(n,n) to ppois(n+1,n)=1−pgamma(n+1,n+1), where we just obtained that the former upper envelope sequence is decreasing in *n*, and based on the result of [1], the latter lower envelope sequence is increasing in *n* (with common limit 1/2 as n→∞).

Clearly, Theorem 2 is equivalent to

$$\alpha \mapsto \mathrm{pbeta}\left(\frac{\alpha -1}{\alpha +\beta -1},\alpha ,\beta \right)$$

increasing for α>1 (it is zero for 0<α≤1), where, in fact, (α−1)/(α+β−1) is the *harmonic mean* of the Beta distribution with parameters α and β. Thus, if for α>max(u,0) and β>0, we write

$$p_{\alpha ,\beta ,u}=\mathrm{pbeta}\left(\frac{\alpha -u}{\alpha +\beta -u},\alpha ,\beta \right),$$

and our results say that $\alpha \mapsto p_{\alpha ,\beta ,0}$ is decreasing, and $\alpha \mapsto p_{\alpha ,\beta ,1}$ is increasing, and we can ask about the monotonicity for other values of *u*. Numerical experiments suggest that for β>1, $\alpha \mapsto p_{\alpha ,\beta ,u}$ is decreasing iff u≤1/3 and increasing iff u≥0.5, but we have thus far been unable to obtain rigorous monotonicity results for u∉{0,1}. We also note also that for u=1, we find that for α>1, the median $m_{\alpha ,\beta }$ of the Beta distribution with parameters α and β satisfies $m_{\alpha ,\beta }>\left(\alpha -1\right)/\left(\alpha +\beta -1\right)$, which for α<β is worse than the lower bound $m_{\alpha ,\beta }>\left(\alpha -1\right)/\left(\alpha +\beta -2\right)$ of [6]. This suggests the importance of more generally investigating the monotonicity of $\alpha \mapsto p_{\alpha ,\beta ,u,v}=\mathrm{pbeta}\left(\left(\alpha -u\right)/\left(\alpha +\beta -v\right),\alpha ,\beta \right)$. For v=2u, this includes the bounds in [6] and the approximations suggested in [7], and it conveniently allows using $p_{\alpha ,\beta ,u,2u}=1-p_{\beta ,\alpha ,u,2u}$ to obtain monotonicity in β from the monotonicity in α.

## 3. Proofs

We first establish several lemmas. We write that

$$g_{\alpha ,\beta }={\left.\frac{x^{\alpha }{\left(1-x\right)}^{\beta }}{\alpha B(\alpha ,\beta )}\right|}_{x=\frac{\alpha }{\alpha +\beta }}=\frac{\alpha^{\alpha -1}\beta^{\beta }}{B\left(\alpha ,\beta \right){\left(\alpha +\beta \right)}^{\alpha +\beta }}$$

**Lemma** **1.** *Let α,β>0. Then,*

$$\mathrm{pbeta}\left(\frac{\alpha }{\alpha +\beta },\alpha ,\beta \right)=\mathrm{pbeta}\left(\frac{\alpha }{\alpha +\beta },\alpha +1,\beta \right)+g_{\alpha ,\beta }.$$

**Proof.**  Immediate from Equation 8.17.20 (http://dlmf.nist.gov/8.17.E20, accessed on 28 October 2024) in [8]. □

**Lemma** **2.** *Let β>0. Then, $\alpha \mapsto g_{\alpha ,\beta }$ is decreasing and positive for α>0.*

**Proof.**  Positivity is trivial. As

$$g_{\alpha ,\beta }=\frac{\beta^{\beta }}{\Gamma (\beta )}\frac{\Gamma (\alpha +\beta )}{\Gamma (\alpha )}\frac{\alpha^{\alpha -1}}{{\left(\alpha +\beta \right)}^{\alpha +\beta }},$$

we have

$$\frac{\partial \log(g_{\alpha ,\beta })}{\partial \alpha }=\psi \left(\alpha +\beta \right)-\psi \left(\alpha \right)+\log\left(\alpha \right)-\frac{1}{\alpha }-\log\left(\alpha +\beta \right),$$

where $\psi \left(z\right)={\left(\log\left(\Gamma \left(z\right)\right)\right)}^{\prime }=\Gamma^{\prime }\left(z\right)/\Gamma \left(z\right)$ is the psi (or digamma) function (e.g., Equation 5.2.2 (https://dlmf.nist.gov/5.2.E2, accessed on 28 October 2024) in [8]). Using Equation 5.9.13 (http://dlmf.nist.gov/5.9.E13, accessed on 28 October 2024) in [8], for ℜ(z)>0, we find that

$$\psi \left(z\right)-\log\left(z\right)=\int_{0}^{\infty }\left(\frac{1}{t}-\frac{1}{1-e^{-t}}\right)e^{-zt}\;dt,$$

so that

$$\begin{array}{ccc} \frac{\partial \log(g_{\alpha ,\beta })}{\partial \alpha } & = & \int_{0}^{\infty }\left(\frac{1}{t}-\frac{1}{1-e^{-t}}\right)\left(e^{-(\alpha +\beta )t}-e^{-\alpha t}\right)\;dt-\frac{1}{\alpha }\\ & = & \int_{0}^{\infty }\left(\frac{1}{t}-\frac{1}{1-e^{-t}}\right)\left(e^{-\beta t}-1\right)e^{-\alpha t}\;dt-\int_{0}^{\infty }e^{-\alpha t}\;dt\\ & = & \int_{0}^{\infty }\left(\left(\frac{1}{t}-\frac{1}{1-e^{-t}}\right)\left(e^{-\beta t}-1\right)-1\right)e^{-\alpha t}\;dt\\ & = & \int_{0}^{\infty }\left(k\left(t\right)\left(1-e^{-\beta t}\right)-1\right)e^{-\alpha t}\;dt, \end{array}$$

where

$$k\left(t\right)=\frac{1}{1-e^{-t}}-\frac{1}{t}$$

has the derivative

$$k^{\prime }\left(t\right)=-\frac{e^{-t}}{{\left(1-e^{-t}\right)}^{2}}+\frac{1}{t^{2}}=\frac{-t^{2}e^{-t}+{\left(1-e^{-t}\right)}^{2}}{t^{2}{\left(1-e^{-t}\right)}^{2}}$$

with

$$-t^{2}e^{-t}+{\left(1-e^{-t}\right)}^{2}=1-2e^{-t}+e^{-2t}-t^{2}e^{-t}=e^{-t}\left(e^{t}-\left(2+t^{2}\right)+e^{-t}\right)>0$$

for t>0. Hence, for all t>0, *k* is increasing, from which first k(t)<k(∞)=1 and then $k\left(t\right)\left(1-e^{-\beta t}\right)-1<1-1=0$ are derived. This in turn shows that $\log(g_{\alpha ,\beta })$ and, hence, $g_{\alpha ,\beta }$ are decreasing for α>0. □

**Lemma** **3.** *Let α,β>0. Then,*

$$\begin{array}{c} \mathrm{pbeta}\left(\frac{\alpha +1}{\alpha +\beta +1},\alpha +1,\beta \right)-\mathrm{pbeta}\left(\frac{\alpha }{\alpha +\beta },\alpha +1,\beta \right)\\ \;=\frac{1}{B(\alpha +1,\beta )}\int_{0}^{1}\frac{{\left(\alpha +v\right)}^{\alpha }\beta^{\beta }}{{\left(\alpha +\beta +v\right)}^{\alpha +\beta +1}}\;dv\\ \;=g_{\alpha +1,\beta }\int_{0}^{1}{\left(\frac{\alpha +v}{\alpha +1}\right)}^{\alpha }{\left(\frac{\alpha +\beta +1}{\alpha +\beta +v}\right)}^{\alpha +\beta +1}\;dv\\ \;=g_{\alpha ,\beta }\int_{0}^{1}{\left(\frac{\alpha +v}{\alpha }\right)}^{\alpha }{\left(\frac{\alpha +\beta }{\alpha +\beta +v}\right)}^{\alpha +\beta +1}\;dv. \end{array}$$

**Proof.**  We have

$$\begin{array}{c} \mathrm{pbeta}\left(\frac{\alpha +1}{\alpha +\beta +1},\alpha +1,\beta \right)-\mathrm{pbeta}\left(\frac{\alpha }{\alpha +\beta },\alpha +1,\beta \right)\\ \;=\frac{1}{B(\alpha +1,\beta )}\int_{\frac{\alpha }{\alpha +\beta }}^{\frac{\alpha +1}{\alpha +1+\beta }}t^{\alpha }{\left(1-t\right)}^{\beta -1}\;dt. \end{array}$$

Substituting t=u/(1+u) so that 1−t=1/(1+u) and $dt=du/{\left(1+u\right)}^{2}$ and then u=(α+v)/β,

$$\begin{array}{ccc} \int_{\frac{\alpha }{\alpha +\beta }}^{\frac{\alpha +1}{\alpha +1+\beta }}t^{\alpha }{\left(1-t\right)}^{\beta -1}\;dt & = & \int_{\frac{\alpha }{\beta }}^{\frac{\alpha +1}{\beta }}\frac{u^{\alpha }}{{\left(1+u\right)}^{\alpha +\beta +1}}\;du\\ & = & \frac{1}{\beta }\int_{0}^{1}{\left(\frac{\alpha +v}{\beta }\right)}^{\alpha }{\left(\frac{\beta }{\beta +\alpha +v}\right)}^{\alpha +\beta +1}\\ & = & \int_{0}^{1}\frac{{\left(\alpha +v\right)}^{\alpha }\beta^{\beta }}{{\left(\alpha +\beta +v\right)}^{\alpha +\beta +1}}\;dv \end{array}$$

from which the first equality follows. The second is immediate, and the third obtained from

$$g_{\alpha ,\beta }=\frac{\alpha^{\alpha }\beta^{\beta }}{{\left(\alpha +\beta \right)}^{\alpha +\beta }}\frac{1}{\alpha B(\alpha ,\beta )}=\frac{\alpha^{\alpha }\beta^{\beta }}{{\left(\alpha +\beta \right)}^{\alpha +\beta +1}}\frac{1}{B(\alpha +1,\beta )}.$$

□

Write

$$h_{\alpha ,\beta }=1-\int_{0}^{1}{\left(\frac{\alpha +v}{\alpha }\right)}^{\alpha }{\left(\frac{\alpha +\beta }{\alpha +\beta +v}\right)}^{\alpha +\beta +1}\;dv.$$

**Lemma** **4.** *Let β>0. Then, $\alpha \mapsto h_{\alpha ,\beta }$ is decreasing and positive for α>0.*

**Proof.**  We write

$$h_{\alpha ,\beta }=1-\int_{0}^{1}k_{\alpha ,\beta }\left(v\right)\;dv,\;k_{\alpha ,\beta }\left(v\right)={\left(\frac{\alpha +v}{\alpha }\right)}^{\alpha }{\left(\frac{\alpha +\beta }{\alpha +\beta +v}\right)}^{\alpha +\beta +1}.$$

Then,

$$\log\left(k_{\alpha ,\beta }\left(v\right)\right)=\alpha \log\frac{\alpha +v}{\alpha }+\left(\alpha +\beta +1\right)\log\frac{\alpha +\beta }{\alpha +\beta +v}$$

and, hence,

$$\begin{array}{ccc} \frac{\partial \log(k_{\alpha ,\beta }\left(v\right))}{\partial \alpha } & = & \log\frac{\alpha +v}{\alpha }+\alpha \left(\frac{1}{\alpha +v}-\frac{1}{\alpha }\right)\\ & & +\log\frac{\alpha +\beta }{\alpha +\beta +v}+\left(\alpha +\beta +1\right)\left(\frac{1}{\alpha +\beta }-\frac{1}{\alpha +\beta +v}\right). \end{array}$$

As a function of *v*, this has the derivative

$$\begin{array}{ccc} \frac{\partial }{\partial v}\frac{\partial \log(k_{\alpha ,\beta }\left(v\right))}{\partial \alpha } & = & \frac{1}{\alpha +v}-\frac{\alpha }{{\left(\alpha +v\right)}^{2}}-\frac{1}{\alpha +\beta +v}+\frac{\alpha +\beta +1}{{\left(\alpha +\beta +v\right)}^{2}}\\ & = & \frac{v}{{\left(\alpha +v\right)}^{2}}+\frac{1-v}{{\left(\alpha +\beta +v\right)}^{2}} \end{array}$$

which is positive for 0<v<1. Hence, for α,β>0 and 0<v<1, so

$$\frac{\partial \log(k_{\alpha ,\beta }\left(v\right))}{\partial \alpha }>{\left.\frac{\partial \log(k_{\alpha ,\beta }\left(v\right))}{\partial \alpha }\right|}_{v=0}=0$$

from which we infer that for β>0 and 0<v<1, $\alpha \mapsto k_{\alpha ,\beta }\left(v\right)$ is increasing for α>0, which in turn yields that $\alpha \mapsto h_{\alpha ,\beta }$ is decreasing for α>0.

For α→∞,

$$k_{\alpha ,\beta }\left(v\right)=\frac{{\left(1+v/\alpha \right)}^{\alpha }}{{\left(1+v/\left(\alpha +\beta \right)\right)}^{\alpha +\beta +1}}\to \frac{e^{v}}{e^{v}}=1$$

and, hence, $h_{\alpha ,\beta }\to h_{\infty ,\beta }=1-\int_{0}^{1}1\;dv=0$. Thus, for all α>0, $h_{\alpha ,\beta }>h_{\infty ,\beta }=0$, thus completing the proof. □

**Lemma** **5.** *Let α,β>0. Then,*

$$p_{\alpha ,\beta }=p_{\alpha +1,\beta }+g_{\alpha ,\beta }h_{\alpha ,\beta }$$

*and*

$$p_{\alpha ,\beta }=p_{\infty ,\beta }+\sum_{n=0}^{\infty }g_{\alpha +n,\beta }h_{\alpha +n,\beta }.$$

**Proof.**  Using Lemmas 1 and 3,

$$\begin{array}{ccc} p_{\alpha ,\beta } & = & \mathrm{pbeta}\left(\frac{\alpha }{\alpha +\beta },\alpha ,\beta \right)\\ & = & \mathrm{pbeta}\left(\frac{\alpha }{\alpha +\beta },\alpha +1,\beta \right)+g_{\alpha ,\beta }\\ & = & \mathrm{pbeta}\left(\frac{\alpha +1}{\alpha +\beta +1},\alpha +1,\beta \right)+g_{\alpha ,\beta }\\ & & -\left(\mathrm{pbeta}\left(\frac{\alpha +1}{\alpha +\beta +1},\alpha +1,\beta \right)-\mathrm{pbeta}\left(\frac{\alpha }{\alpha +\beta },\alpha +1,\beta \right)\right)\\ & = & p_{\alpha +1,\beta }+g_{\alpha ,\beta }\left(1-\int_{0}^{1}{\left(\frac{\alpha +v}{\alpha }\right)}^{\alpha }{\left(\frac{\alpha +\beta }{\alpha +\beta +v}\right)}^{\alpha +\beta +1}\;dv\right)\\ & = & p_{\alpha +1,\beta }+g_{\alpha ,\beta }h_{\alpha ,\beta }, \end{array}$$

from which

$$p_{\alpha ,\beta }=p_{\infty ,\beta }+\sum_{n=0}^{\infty }\left(p_{\alpha +n,\beta }-p_{\alpha +n+1,\beta }\right)=p_{\infty ,\beta }+\sum_{n=0}^{\infty }g_{\alpha +n,\beta }h_{\alpha +n,\beta }$$

as asserted. □

**Proof** **of Theorem 1.** As x↦t(x)=x/(1+x) increases monotonically from 0 to 1 as *x* varies from 0 to *∞*, and t(u/v)=u/(u+v),

$$p_{\alpha ,\beta }=P\left(t\left(X_{\alpha }/X_{\beta }\right)\leq t\left(\alpha /\beta \right)\right)=P\left(\frac{X_{\alpha }}{X_{\alpha }+X_{\beta }}\leq \frac{\alpha }{\alpha +\beta }\right)$$

where $Y_{\alpha ,\beta }=X_{\alpha }/\left(X_{\alpha }+X_{\beta }\right)∼\mathrm{Beta}\left(\alpha ,\beta \right)$ has the mean $E\left(Y_{\alpha ,\beta }\right)=\alpha /\left(\alpha +\beta \right)$, so that $p_{\alpha ,\beta }=\mathrm{pbeta}\left(\alpha /\left(\alpha +\beta \right),\alpha ,\beta \right)$.

Combining Lemmas 2, 4 and 5, we see that $\alpha \mapsto p_{\alpha ,\beta }$ is decreasing for α>0. To determine the limits, remember that if $X_{\alpha }∼\mathrm{Gamma}\left(\alpha \right)$, then $X_{\alpha }/\alpha$ goes to 0 in probability as α→0+ and to 1 as α→∞. Hence,

$$p_{\alpha ,\beta }=P\left(\frac{X_{\alpha }}{\alpha }\leq \frac{X_{\beta }}{\beta }\right)$$

goes to $P(0\leq X_{\beta }/\beta )=1$ as α→0+ and to $P\left(1\leq X_{\beta }/\beta \right)=1-\mathrm{pgamma}\left(\beta ,\beta \right)$ as α→∞.

Finally, $p_{\beta ,\alpha }=1-p_{\alpha ,\beta }$, thus completing the proof. □

We write

$${\tilde{h}}_{\alpha ,\beta }=\int_{0}^{1}{\left(\frac{\alpha +v}{\alpha +1}\right)}^{\alpha }{\left(\frac{\alpha +\beta +1}{\alpha +\beta +v}\right)}^{\alpha +\beta +1}\;dv-1.$$

**Lemma** **6.** *Let β>0. Then, $\alpha \mapsto {\tilde{h}}_{\alpha ,\beta }$ is decreasing and positive for α>0.*

**Proof.**  This parallels the proof of Lemma 4. We write

$${\tilde{h}}_{\alpha ,\beta }=\int_{0}^{1}{\tilde{k}}_{\alpha ,\beta }\left(v\right)\;dv-1,\;{\tilde{k}}_{\alpha ,\beta }\left(v\right)={\left(\frac{\alpha +v}{\alpha +1}\right)}^{\alpha }{\left(\frac{\alpha +\beta +1}{\alpha +\beta +v}\right)}^{\alpha +\beta +1}.$$

Then,

$$\log\left({\tilde{k}}_{\alpha ,\beta }\left(v\right)\right)=\alpha \log\frac{\alpha +v}{\alpha +1}+\left(\alpha +\beta +1\right)\log\frac{\alpha +\beta +1}{\alpha +\beta +v}$$

and, hence,

$$\begin{array}{ccc} \frac{\partial \log({\tilde{k}}_{\alpha ,\beta }\left(v\right))}{\partial \alpha } & = & \log\frac{\alpha +v}{\alpha +1}+\alpha \left(\frac{1}{\alpha +v}-\frac{1}{\alpha +1}\right)\\ & & +\log\frac{\alpha +\beta +1}{\alpha +\beta +v}+\left(\alpha +\beta +1\right)\left(\frac{1}{\alpha +\beta +1}-\frac{1}{\alpha +\beta +v}\right). \end{array}$$

As a function of *v*, this (again) has the derivative

$$\begin{array}{ccc} \frac{\partial }{\partial v}\frac{\partial \log({\tilde{k}}_{\alpha ,\beta }\left(v\right))}{\partial \alpha } & = & \frac{1}{\alpha +v}-\frac{\alpha }{{\left(\alpha +v\right)}^{2}}-\frac{1}{\alpha +\beta +v}+\frac{\alpha +\beta +1}{{\left(\alpha +\beta +v\right)}^{2}}\\ & = & \frac{v}{{\left(\alpha +v\right)}^{2}}+\frac{1-v}{{\left(\alpha +\beta +v\right)}^{2}} \end{array}$$

which is positive for 0<v<1. Hence, for α,β>0 and 0<v<1,

$$\frac{\partial \log({\tilde{k}}_{\alpha ,\beta }\left(v\right))}{\partial \alpha }<{\left.\frac{\partial \log({\tilde{k}}_{\alpha ,\beta }\left(v\right))}{\partial \alpha }\right|}_{v=1}=0$$

from which we infer that for β>0 and 0<v<1, $\alpha \mapsto {\tilde{k}}_{\alpha ,\beta }\left(v\right)$ is decreasing for α>0, which in turn implies that $\alpha \mapsto {\tilde{h}}_{\alpha ,\beta }$ is decreasing for α>0. Finally, for α→∞,

$${\tilde{k}}_{\alpha ,\beta }\left(v\right)={\left(\frac{1+v/\alpha }{1+1/\alpha }\right)}^{\alpha }{\left(\frac{1+1/(\alpha +\beta )}{1+v/(\alpha +\beta )}\right)}^{\alpha +\beta +1}\to \frac{e^{v}}{e}\frac{e}{e^{v}}=1$$

and, hence, ${\tilde{h}}_{\alpha ,\beta }\to {\tilde{h}}_{\infty ,\beta }=\int_{0}^{1}1\;dv-1=0$. Thus, for all α>0, ${\tilde{h}}_{\alpha ,\beta }>{\tilde{h}}_{\infty ,\beta }=0$, thus completing the proof. □

**Lemma** **7.** *Let α,β>0. Then,*

$${\tilde{p}}_{\alpha ,\beta }={\tilde{p}}_{\alpha +1,\beta }-g_{\alpha +1,\beta }{\tilde{h}}_{\alpha ,\beta }$$

*and*

$${\tilde{p}}_{\alpha ,\beta }={\tilde{p}}_{\infty ,\beta }-\sum_{n=0}^{\infty }g_{\alpha +n+1,\beta }{\tilde{h}}_{\alpha +n,\beta }.$$

**Proof.**  Using Lemma 1 (with α+1 instead of α) and Lemma 3, we have

$$\begin{array}{ccc} {\tilde{p}}_{\alpha ,\beta } & = & \mathrm{pbeta}\left(\frac{\alpha }{\alpha +\beta },\alpha +1,\beta \right)\\ & = & \mathrm{pbeta}\left(\frac{\alpha +1}{\alpha +1+\beta },\alpha +1,\beta \right)\\ & & -\left(\mathrm{pbeta}\left(\frac{\alpha +1}{\alpha +\beta +1},\alpha +1,\beta \right)-\mathrm{pbeta}\left(\frac{\alpha }{\alpha +\beta },\alpha +1,\beta \right)\right)\\ & = & \mathrm{pbeta}\left(\frac{\alpha +1}{\alpha +1+\beta },\alpha +2,\beta \right)+g_{\alpha +1,\beta }\\ & & -g_{\alpha +1,\beta }\int_{0}^{1}{\left(\frac{\alpha +v}{\alpha +1}\right)}^{\alpha }{\left(\frac{\alpha +\beta +1}{\alpha +\beta +v}\right)}^{\alpha +\beta +1}\;dv\\ & = & {\tilde{p}}_{\alpha +1,\beta }-g_{\alpha +1,\beta }{\tilde{h}}_{\alpha ,\beta }. \end{array}$$

The second assertion again follows by taking telescope sums. □

**Proof** **of Theorem 2.** Combining Lemmas 2, 6 and 7, we see that $\alpha \mapsto {\tilde{p}}_{\alpha ,\beta }$ is increasing for α>0. The limits are immediate from (the proof of) Theorem 1. □

## Data Availability

The original contributions presented in the study are included in the article, further inquiries can be directed to the corresponding author.
